# Modeling Epilepsy Using Human Induced Pluripotent Stem Cells-Derived Neuronal Cultures Carrying Mutations in Ion Channels and the Mechanistic Target of Rapamycin Pathway

**DOI:** 10.3389/fnmol.2022.810081

**Published:** 2022-03-10

**Authors:** Octavia Yifang Weng, Yun Li, Lu-Yang Wang

**Affiliations:** ^1^Program in Developmental and Stem Cell Biology, Sick Kids Research Institutes, Toronto, ON, Canada; ^2^Program in Neuroscience and Mental Health, Sick Kids Research Institutes, Toronto, ON, Canada; ^3^Department of Physiology, University of Toronto, Toronto, ON, Canada; ^4^Department of Molecular Genetics, University of Toronto, Toronto, ON, Canada

**Keywords:** epilepsy, iPSC, ion channel, mTOR signaling, homeostasis

## Abstract

Epilepsy is a neurological disorder that affects over 65 million people globally. It is characterized by periods of seizure activity of the brain as a result of excitation and inhibition (E/I) imbalance, which is regarded as the core underpinning of epileptic activity. Both gain- and loss-of-function (GOF and LOF) mutations of ion channels, synaptic proteins and signaling molecules along the mechanistic target of rapamycin (mTOR) pathway have been linked to this imbalance. The pathogenesis of epilepsy often has its roots in the early stage of brain development. It remains a major challenge to extrapolate the findings from many animal models carrying these GOF or LOF mutations to the understanding of disease mechanisms in the developing human brain. Recent advent of the human pluripotent stem cells (hPSCs) technology opens up a new avenue to recapitulate patient conditions and to identify druggable molecular targets. In the following review, we discuss the progress, challenges and prospects of employing hPSCs-derived neural cultures to study epilepsy. We propose a tentative working model to conceptualize the possible impact of these GOF and LOF mutations in ion channels and mTOR signaling molecules on the morphological and functional remodeling of intrinsic excitability, synaptic transmission and circuits, ultimately E/I imbalance and behavioral phenotypes in epilepsy.

Seizure is the result of a temporary disruption in neuronal activity due to excessive synchrony of neurons. Epilepsy is a chronic condition during which recurrent periods of seizures take place and is sometimes accompanied by comorbidities such as developmental and intellectual delay, depression, anxiety, heart disease, and others. Epilepsy is diagnosed based on the abnormal electrical activity and brain structure underlying the hypersynchrony in neurons. Neuroimaging techniques such as electroencephalogram (EEG), magnetic resonance imaging (MRI), positron emission tomography (PET), and single-photon emission computerized tomography (SPECT) have all been used for diagnosis. Currently, epilepsy is categorized into four types, including generalized epilepsy, focal epilepsy, generalized and focal epilepsy, and unknown epilepsy. In generalized epilepsy, seizures start on both sides of the brain, leading to absence epilepsy with little to no movement or tonic-clonic epilepsy with stiffening or jerking movements. Myoclonic epilepsy involves local or global muscles contraction whilst atonic epilepsy is associated with a sudden loss of consciousness, both of which have been observed in generalized epilepsy. In contrast, seizures in focal epilepsy start at a confined region in the brain, and the patient symptoms usually depend on the region affected. In this type of epilepsy, patients can be awake or unconscious (Stafstrom and Carmant, 2015). The third type involves the combination of focal and generalized epilepsy, where the seizures can start either locally or globally in the patients. When the clinical diagnosis fails to ascribe symptoms to focal or generalized epilepsy, the patient is assigned to the last category, unknown epilepsy.

Genetic mutations, *de novo* or inherited, play a major role in the pathogenesis of epilepsy. Among various forms of epilepsy, mutations in ion channels and mTOR signaling molecules lead to converging epileptic types, ranging from focal to generalized epilepsy (Mulley et al., 2003; Perucca, 2018; Proietti Onori et al., 2021). Many of the genetic mutations underlying channelopathies directly affect the intrinsic excitability of neurons and/or synaptic transmission (Mulley et al., 2003; Lascano et al., 2016; Bartolini et al., 2020), while other mutations along the mTOR signaling pathway can indirectly affect the level and activity of ion channels that determine neuronal firing rate, patterns and network activity (Cho, 2011). Epilepsy can be caused by structural changes in the brain, which could be congenital or acquired later in life through external means such as brain trauma or stroke, and epilepsy itself can also promote aberrant neuronal growth known as sprouting (Scharfman, 2007; Cho, 2011). Other factors such as changes in the metabolic and immune environment in the brain may increase the propensity of epilepsy (Patel, 2018; Xu et al., 2018). In this review, we will focus on epilepsy primarily stemming from genetic mutations.

Epileptic pathogenesis has been linked to excitatory/inhibitory (E/I) imbalance, where the excitatory and inhibitory input ratio is pathologically altered. E/I balance can be defined as a singular entity at a global circuit level, where it has been shown to affect brain states, such as an increase in inhibition ratio in awake over the anesthetized state (Haider et al., 2013). At a single neuron level, E/I balance can be shaped by intrinsic neuronal excitability from various ion channels and the interplay of excitatory and inhibitory synaptic inputs, typically glutamatergic or GABAergic signals, defining wiring and firing property in normal development. In epilepsy, changes in ion channels, synaptic inputs, and morphology such as activity-induced neuronal sprouting lead to misfiring and miswiring in a vicious cycle, underlying a progressively hypersynchronous brain circuit (Cavarsan et al., 2018). While disruption of short-term E/I balance can lead to hyperexcitation underlying seizure activity, the chronic nature of epilepsy development may need cooperative changes to remodel the homeostatic setpoint which is influenced by many factors. For example, mitochondrial dihydroorotate dehydrogenase (DHODH) (Styr et al., 2019; Ruggiero et al., 2021) has been shown to be one of the key determinants of the homeostatic setpoint as inhibition of DHODH can reduce firing rate setpoint and the susceptibility to seizures.

Rodent models have been widely used to study epilepsy. Conventional models include audiogenic seizure susceptible DBA/2 mouse or genetic *absence* epilepsy rat from Strasbourg (GAERS), in which many antiepileptic drugs (AEDs) were developed (Löscher, 2017). More recently, genetically engineered animal models have been developed by introducing patient mutations into animals to mimic the human disease conditions (Hirose et al., 2020). These studies have built an essential groundwork for the understanding of epilepsy mechanisms and the exploration of therapeutic solutions. Some animal models recapitulate seizure activity well, such as those with SCN1A mutation for Dravet syndrome (Ogiwara et al., 2007). However, other mouse models, such as those with disease-relevant KCNQ2 mutations, were unable to reproduce the spontaneous seizure phenotypes seen in human patients (Ihara et al., 2016; Hirose et al., 2020). In addition, AEDs that are effective in animal models often fail to do so in human patients, suggesting that species difference may be an important factor that influences the effectiveness of treatment, especially for patients with intractable epilepsy (Löscher and Schmidt, 2011). These challenges highlight the pressing need to build a continuum of models from laboratory animals to human subjects.

Since the introduction of induced pluripotent stem cells (iPSCs) in 2007 (Takahashi and Yamanaka, 2006; Takahashi et al., 2007), the generation and the biobanking of patient-specific pluripotent stem cell lines have opened up a new avenue to study human diseases. It is now possible to gain mechanistic understandings of the genesis and pathologies associated with epilepsy, and to investigate suitable treatment options for individual patients. Combined with an ongoing revolution in the gene editing field, such as the CRISPR/Cas9 technology, human *in vitro* model systems represent an unprecedented opportunity to capture the clinical conditions and further apprehend the underpinnings of a variety of other genetic disorders including epilepsy. With the insights from previous animal models and the rapid technological developments in stem cells and genetic engineering, we now have scalable tools to study the spatiotemporal nature of epileptic pathogenesis. Specifically, by mapping out where aberrant activity initiates, how the original epileptic loci recruit neighboring neurons and the temporal stage at which such phenotypes appear, we will gain in-depth knowledge into epilepsy pathogenesis and identify new molecular substrates for potential therapeutics. This review will examine recent advancements in human *in vitro* studies on epilepsy caused by different genetic mutations, followed by prospective discussions on the key issues that can further advance the mechanistic understanding of epilepsy.

## Modeling Channelopathy in Epilepsy With Human *in vitro* System

Channelopathy accounts for the majority of genetic mutations associated with epilepsy. The most common ion channel mutated in epilepsy include voltage-gated sodium, potassium, calcium channels, ligand-gated glutamatergic and GABAergic receptors (Oyrer et al., 2018). Although various ion channels mutations reported from clinical data have been studied in animal models, only a few have been studied using the human *in vitro* systems.

### Voltage-Gated Sodium Channels

Voltage-gated sodium channels (Nav) play an essential role in the depolarizing phase of the action potential (or spike), promoting spike firings. In the mature central nervous system of mammals, Nav1.1, Nav1.2, and Nav1.6 (encoded by SCN1A, SCN2A, and SCN8A, respectively) are the most abundant. While all three are linked to epilepsy, only Nav1.1 has been extensively studied in the human *in vitro* system. Nav1.1 is located on the axon initiation site (AIS) of neurons and contributes to the initiation and propagation of action potentials as well as their excitability (Child and Benarroch, 2014). Using epilepsy patient iPSCs-derived inhibitory neurons, it has been shown that neurons carrying a loss-of-function (LOF) SCN1A mutation (S1328P) displayed a reduction in Na^+^ current amplitude (Sun et al., 2016) (Table 1). SCN1A (c.4261G>T/c.3576_3580del TCAAA) mutated neurons exhibited decreased Na^+^ current density (Kim et al., 2018), with SCN1A (c. 4261G > T) showing more reduction in Na^+^ current compared to SCN1A (c.3576_3580del TCAAA). The differences in biophysical properties from these mutations also matched with the symptom severity in the patients whose iPSCs were generated from. Interestingly, the changes in electrical activity were mainly observed with inhibitory neurons but not excitatory neurons (Sun et al., 2016). In contrast, a gain-of-function (GOF) effect from SCN1A (F1415I) or SCN1A (Q1923R) in excitatory neurons with hyperexcitability has also been reported (Jiao et al., 2013). This discrepancy led to a series of follow-up studies clarifying the role of Nav1.1 mutation in inhibitory neurons and excitatory neurons. One possible explanation was that the mutations and the genetic background of the patient in the studies were different. To rule out the interference of the genetic background, genetically engineered isogenic control and mutant cells carrying patient-specific mutation SCN1A (Q1923R) were generated (Liu et al., 2016). Mutant inhibitory neurons displayed a decrease in Na^+^ current density, leading to reduced amplitude and number of action potential in response to the same magnitude of current injections. In addition, frequency and amplitude of spontaneous inhibitory postsynaptic currents (sIPSCs) in the inhibitory neurons decreased, indicating that lowered excitability in these neurons likely attenuates inhibitory output onto other cells to elevate E/I ratio. Furthermore, both inhibitory and excitatory neurons derived from patient cells or CRISPR/Cas9 engineered iPSCs carrying the same patient mutation SCN1A (K1270T) confirmed convergent phenotypes in inhibitory neurons, including decreased action potential frequency, amplitude, and Na^+^ current density. In contrast, decreased Na^+^ current density was reported in excitatory neurons, paradoxically, with an increase in firing frequency. The heightened frequency may result from the mutation and lead to a broader voltage range for sodium channel openings (Xie et al., 2020). These findings demonstrate the importance of LOF mutations in inhibitory neurons, but do not rule out the possibility of GOF mutations with a different genetic background targeting excitatory neurons as previously reported. Future experiments on co-culture of both excitatory and inhibitory neurons to directly measure E/I ratio with the same genetic background is clearly needed to draw a firm conclusion on the roles of SCN1A LOF and GOF mutations in epilepsy. Although the literature has largely focused on SCN1A, cell lines derived from epilepsy patients with SCN2A and SCN8A LOF mutations have been developed for future studies (Tidball et al., 2017).

**TABLE 1:** Summary of human iPSC-derived cultures on epilepsy related to ion channels and mTOR pathway mutations.

Gene	Epileptic syndrome	Morphological and electrical characterizations	Model
**Ion channel**			
SCN1A	Dravet’s syndrome	Increased excitability and Na^+^ current in excitatory neurons	Patient (F1415I/Q1923R) iPSCs derived excitatory neurons (Jiao et al., 2013)
SCN1A	Dravet’s syndrome	Decreased Na^+^ current and AP in inhibitory neurons, but not in excitatory neurons	Patient (S1328P) iPSCs derived inhibitory and excitatory neurons (Sun et al., 2016)
SCN1A	Dravet’s syndrome	Decreased Na^+^ current density and lowered AP amplitude and number in current clamp; lower frequency and amplitude of sIPSCs in inhibitory neurons	Patient (Q1923R) iPSCs derived inhibitory neurons (Liu et al., 2016)
SCN1A	Dravet’s syndrome	Decreased number of AP and Na^+^ current density in derived GABAergic neurons	Patient (c.4261G > T/c.3576_3580del TCAAA) iPSCs derived inhibitory neurons (Kim et al., 2018)
SCN1A	Dravet’s syndrome	Decreased AP frequency, amplitude, and Na^+^ current density in inhibitory neurons; decreased Na^+^ current density but increased firing frequency in excitatory neurons	Patient (K1270T) iPSCs or CRISPR/Cas9 engineered iPSCs carrying patient mutations derived inhibitory or excitatory neurons (Xie et al., 2020)
KCNQ2	Neonatal epileptic encephalopathy	Increased bursting firing with faster AP repolarization and shorter AP half width	Patient (c.1742G > A) iPSCs derived excitatory neurons (Simkin and Kiskinis, 2018)
KCNT1	Malignant migrating partial seizures of infancy	Shorter AP, increased KNa^+^ current, increased afterhyperpolarization amplitude	Patient (P924L) iPSCs derived neurons (mixture of glutamatergic and GABAergic neurons) (Quraishi et al., 2019)
**mTOR pathway**			
TSC1/2	Tuberous sclerosis	Increased proliferation rate in neural stem cells	Patient (c.1444-2A > C) iPSCs derived neural stem cells (Li et al., 2017)
TSC1/2	Tuberous sclerosis	Enlarged soma size and altered neurite length; elevated network activity, with increased synchrony and mean firing rate	Patient (c.5238_5255del) iPSCs derived neurons (Winden et al., 2019)
TSC1/2	Tuberous sclerosis	Increased frequency of calcium influx and spontaneous spikes	Patient (c.2249G > A/c.1563dupA) iPSCs derived neurons (Nadadhur et al., 2019)
TSC1/2	Tuberous sclerosis	Reduced firing frequency and mEPSCs frequency	Patient iPSCs (Chr16:2088303-2088320_del 18bp/Chr16:2088299-2088306_del8bp) derived cerebellar Purkinje neurons (Sundberg et al., 2018)
TSC1/2	Tuberous sclerosis	Increased soma size and dendritic arborization; lower input resistance, decreased mEPSCs and sEPSCs frequency	Zinc-finger nuclease-mediated targeted gene targeting exon 11 of TSC2 gene in hESCs cell line differentiated into neurons (Costa et al., 2016)
DEPDC5	Familial focal epilepsy	Enlarged soma size; altered mTOR signaling rescued by rapamycin	Patient (c.2620C > T; p.R874*/c.59-493_146 + 710; p.D20Afs*25) iPSCs derived neurons (Klofas et al., 2020)
CDKL5	CDKL5 deficiency disorder	Reduced proliferation rate and increased death in neural progenitor cells; increased dendritic length, higher complexity, and increased hyperexcitability with more number of evoked AP, elevated sEPSC frequency and increased firing rate and synchrony in neurons	Patient (R59X/R550X/S855X/R59X/p.D135_F154del/Xp22.13del) iPSCs derived neural progenitor cells and neurons (Negraes et al., 2021)
UBE3A	Angelman syndrome	Fewer Ca^2+^ transients, decreased AP amplitude, AP threshold, and elevated AP width at later development time point (week 20 and later); reduced frequency of spontaneous currents when induced for LTP	CRISPR/Cas9 edited iPSCs-derived neurons to knockout UBE3A with non-homologous end joining or knockdown UBE3A with antisense oligonucleotides (Fink et al., 2017)
UBE3A	15q11-q13 duplication syndrome	Increased number of dendritic protrusions, heightened excitatory synaptic current frequency and amplitude, lowered inhibitory synaptic currency and amplitude. Disrupted ion channel (KCNQ2) function, impaired activity dependent plasticity, and synaptic scaling	15q11-13 patient iPSCs-derived neurons (Fink et al., 2021)
MECP2	Rett syndrome	Enlarged soma size and increased dendritic branching; reduced protein synthesis and translation	TALEN edited hESCs-derived neurons targeting third exon of MECP2 gene (Li et al., 2013)

### Potassium Channels

Potassium channels comprise the largest group of ion channels. Voltage-gated potassium channels (Kv) allow selective efflux of potassium ions, regulating neuronal spike waveform and firing patterns by repolarizing and hyperpolarizing membrane potentials to prevent hyperactivity and aberrant firings. Numerous mutations have been linked to epilepsy, but only a few, such as Kv7.2 and KNa1.1, have been modeled in the human *in vitro* system. A study investigated KCNQ2, encoding Kv7.2 mediating a current called M-current, which lowers the excitability of neurons and limits repetitive firings. The authors observed that KCNQ2 LOF mutation (c.1742G > A) led to faster action potential repolarization and shorter spike width (Simkin and Kiskinis, 2018). With a loss of Kv7.2 activity, the mutant neurons showed higher spontaneous firing frequency and burst activity than the control as revealed by a multi-electrode array (MEA) assay. This showed that LOF mutation of KCNQ2 in excitatory neurons led to an increased excitability. Several studies have established cell lines from patients with KCNA2 LOF mutations as the tools for future studies (Schwarz et al., 2018, 2019; Uysal et al., 2019). In addition, a GOF mutation in KCNT1, encoding for a sodium-activated potassium channel KNa1.1, has been reported in patients with focal epilepsy. Patient-derived neurons with KCNT1 (P924L) mutation showed hyperexcitability with narrower spike width, higher spontaneous firing rate burst activity and synchronized discharge of the network, though it is unknown if excitatory or inhibitory or both neurons express KCNT1 mutation (Quraishi et al., 2019).

Epilepsy is a multicomponent disease, in which genetic mutations of many types of ion channels from human patients have been implicated. These include hyperpolarization-activated cyclic nucleotide-gated (HCN) channels and calcium channels, which have not yet been modeled using the human *in vitro* model. E/I balance and maintenance of the neuronal network are critically dependent on ion channels. Channelopathies result in dysregulated intrinsic excitability of excitatory and/or inhibitory neurons and their outputs, and consequentially an E/I imbalance, as exemplified by either higher excitability (e.g., LOF mutation in KCNQ2 or GOF mutation in KCNT1) or lowered inhibition (e.g., LOF mutation in SCN1A). In this regard, *in vitro* human models such as patient-derived neurons, mutations in ion channels, complemented with CRISPR/Cas9 technology, have provided important mechanistic insights into how ion channelopathy, especially in sodium and potassium channels, could affect action potential waveform and firing patterns of the neurons, contributing to E/I imbalance underlying epileptic activity.

## Human *in vitro* Model Recapitulating Epilepsy Caused by the Mechanistic Target of Rapamycin Pathway Mutations

Another cluster of genes often found mutated in epilepsy patients are those encoding components of the mTOR pathway, an essential regulator of cell metabolism and physiology. Upon binding of growth factors, phosphoinositide-3-kinase (PI3K) is activated, which converts phosphatidylinositol (4,5)-bisphosphate (PIP2) to phosphatidylinositol (3,4,5)-triphosphate (PIP3). PIP3 serves as a docking site for AKT, a serine/threonine kinase. When phosphorylated, AKT is released into the cytoplasm and inhibits the tuberous sclerosis protein complex (TSC), which acts as an inhibitor for mTOR thereby controlling cell proliferation, cell growth, and cell survival. Previous clinical and *in vitro* studies have revealed the importance of the mTOR pathway in epileptic pathogenesis, where mutations of key signaling molecules along the mTOR pathway were associated with brain malformations and seizures activity, including but not limited to PTEN, PI3K, AKT3, TSC1/2, DEPDC5, and mTOR (Griffith and Wong, 2018).

### Tuberous Sclerosis Complex

Previous studies have targeted tuberous sclerosis complex (TSC1 and TSC2) to model tuberous sclerosis, a multi-organ disorder characterized by the growth of non-cancerous tumors. TSC1/2 serves as a direct inhibitor of mTORC1, and the loss of TSC1/2 leads to hyperactivation of this pathway, affecting various downstream signaling cascades. Although non-cancerous growing mass is the most common presenting symptom in tuberous sclerosis, 80–90% of patients develop epilepsy along the course of the disease (Thiele, 2004). Animal models with TSC1 mutation revealed neurological deficits such as changes in myelination, enlarged neurons, and development of epilepsy (Meikle et al., 2007; Zeng et al., 2008). Mice with TSC2 inactivation mutation also exhibited neurological deficits, including megacephaly and epilepsy (Zeng et al., 2011). Although TSC1/2 has been studied in mouse models, its role in human neurons has only been investigated recently using human *in vitro* models. Human tuberous sclerosis patient-derived neuroepithelial cells carrying TSC2 mutations displayed increased proliferation rate (Li et al., 2017), and mutant cortical neurons developed enlarged soma and altered neurite length (Winden et al., 2019). Functional analysis revealed an overall hyperexcitability coinciding with increased frequency of calcium oscillations and spontaneous spikes (Nadadhur et al., 2019). These patient-derived cortical neurons formed a network with elevated activity, shown by increased firing rate and synchrony (Winden et al., 2019). In contrast, a different study reported lower input resistance, decreased frequency in miniature and spontaneous excitatory postsynaptic currents (mEPSCs and sEPSCs) in neurons derived from ESCs carrying TSC2 mutations (Costa et al., 2016). In addition, human iPSCs-derived cerebellar Purkinje neurons with TSC1/2 mutation showed reduced firing frequency upon current injection and reduced mEPSCs frequency (Sundberg et al., 2018). These inconsistent results of firing frequency from cortical neurons with TSC2 mutation could be due to the interference from the patient genetic background. Alternatively, as Purkinje neurons project to GABAergic outputs, reduced excitatory synaptic activity to these neurons can be transformed to the disinhibition of downstream neurons, leading to hyperexcitable networks underlying epilepsy.

### DEP Domain-Containing Protein 5

DEP domain-containing protein 5 (DEPDC5) encodes a protein within the GATOR1 complex that negatively regulates the mTOR pathway, and its mutation is commonly found in patients with familial focal epilepsy. As germline homozygous DEPDC5 knockout is embryonic lethal, animal studies used conditional knockout targeting specific brain regions or heterozygous DEPDC5 mutants and reported cell morphological change and seizure activity (Yuskaitis et al., 2018). Similarly, human neural progenitor cells (NPCs) with heterozygous LOF DEPDC5 mutation from epilepsy patients iPSCs displayed elevated mTOR signaling reflected by S6 phosphorylation and larger soma size, which were reversed by rapamycin treatment. Mutant neurons also showed elevated responses to amino acids deprivation which is regulated by the mTOR signaling pathway (Klofas et al., 2020). However, it remains unknown whether and how DEPDC5 mutation impacts firing and wiring that account for the pathologic defects in epilepsy.

### Cyclin-Dependent Kinase-Like 5, Ubiquitin-Protein Ligase E3A, and Methyl CpG Binding Protein

While TSC1/2 and DEPDC5 directly regulate the mTOR pathway, other neurodevelopmental disorder genes such as cyclin-dependent kinase-like 5 (CDKL5), ubiquitin-protein ligase E3A (UBE3A), and methyl CpG binding protein (MECP2) are also known to affect or be affected by the mTOR signaling. Although the exact mechanisms of how these genes are linked to the mTOR signaling are yet to be clarified, patients with CDKL5, UBE3A, and MECP2 mutations often develop epilepsy and display altered mTOR activity (Sun et al., 2018; Negraes et al., 2021). CDKL5 is a gene causally linked to CDKL5 deficiency disorder (CDD), where patients develop intellectual delay and epilepsy. CDKL5 was previously found to alter the expression of components of the mTOR signaling and other synaptic elements in mice, possibly leading to changes in neuronal circuitry and excitability (Schroeder et al., 2019). CDKL5 mutant mouse model has been established, although some human patients’ phenotypes were not captured in the mice model, including the lack of spontaneous seizures in young mice (Negraes et al., 2021). By deriving iPSCs from CDD patient fibroblasts, and then differentiating them into neural precursors, the authors observed reduced proliferation and increased cell death. Neurons derived from CDD patient iPSCs displayed altered morphology and electrical activity. Specifically, neurons with CDKL5 LOF mutation showed increased dendritic length and higher complexity when compared to control neurons. In addition, CDD neurons exhibited hyperexcitability, characterized by an increased number of evoked action potentials and elevated frequency of sEPSCs. The authors also reported an upregulation in the co-association between synaptic proteins in CDD neurons, including mGluR5 and Homer, and GluR1 and GluR2 (Negraes et al., 2021). The alterations in glutamate receptor composition can largely affect synaptic strength of excitatory inputs in these neurons. Interestingly, this observation of hyperexcitability was accompanied by decreased Synapsin 1 and PSD95 density. The CDD neurons also showed increased phosphorylation of molecules in the mTOR pathway, including RPTOR and LARP1, and less responsive feedback to the removal of amino acids, which is regulated by the mTOR pathway. CDD cerebral organoids generated from mutant cells revealed convergent findings as those observed in the monolayer neuronal culture, including aberrant electrical activity, increase in mean firing rate and synchrony consistent with epileptic phenotypes.

Another neurodevelopment disorder related to epilepsy is Angelman syndrome (AS), caused by mutations in the UBE3A gene. Patients with AS usually display intellectual disability, developmental delays and seizures. The link between mTOR and AS was minimally examined in human neurons, although a mouse study revealed that the mTOR complexes played a critical role in AS, where the UBE3A mutant mice displayed increased TSC2 inhibition and hyperactivated mTOR signaling, resulting in motor dysfunction. The effect was rescued by rapamycin treatment (Sun et al., 2015). The link between UBE3A and epilepsy or alteration in the mTOR pathway has not been extensively studied in human *in vitro* models until recently. UBE3A mutant human neurons, generated by CRISPR-mediated knockout or antisense oligonucleotide-mediated knockdown, showed a more depolarized resting membrane potential (Fink et al., 2017). These human AS neurons also contained a lower proportion of cells with mature spike waveform and firing patterns. Action potential threshold and amplitude were reduced while the spike width was broadened. Fewer calcium transients were observed at later development time points (week 20). In contrast to the control neurons with the frequency of sEPSCs being readily upregulated by the stimulation paradigm for long-term potentiation, AS neurons were irresponsive to the same stimulation. Another recent study investigated 15q11-q13 duplication syndrome, a neurodevelopmental disorder related to epilepsy. UBE3A resides within the duplicated region and is thought to contribute to disease pathology. This study found that 15q11-q13 duplication human neurons had overall heightened neuronal excitability and excitatory neurotransmission but reduced inhibitory neurotransmission, tipping the E/I balance. Similar to findings in the AS study, activity-dependent plasticity was absent in the 15q11-q13 duplication neurons (Fink et al., 2021). Together these findings demonstrate disrupted electrophysiological properties and plasticity primarily in excitatory neurons carrying UBE3A patient mutations.

MECP2, a global transcriptional repressor, has a vital role in regulating gene expression and chromatin stability. Patients with mutations in MECP2 develop Rett syndrome, a neurodevelopmental disorder characterized by intellectual delays, progressive loss of motor skills and speech, and seizures. Clinical data has suggested a link between MECP2 and mTOR, as elevated mTOR phosphorylation and increased P70S6K has been reported in Rett syndrome patient brains (Olson et al., 2018). Human cortical neurons derived from gene-edited, isogenic MECP2 mutant human embryonic stem cells (hESCs) developed smaller soma, less complex dendritic arborization, and reduced electric activity. These changes were found to coincide with impaired protein synthesis and reduced mTOR pathway activity. Both morphological phenotypes and protein synthesis defects in the mutant neurons were rescued with genetic activation of the mTOR pathway (Li et al., 2013), highlighting the possible role of the mTOR pathway in Rett Syndrome.

## Homeostasis and Genetic Epilepsy

### Synaptic Homeostasis in Neuronal Circuit

The term homeostasis was first coined in 1865 by Claude Bernard as “the stability of the internal environment.” In general, homeostasis involves maintaining the balance of a physiological state or the biological systems through self-regulating mechanisms. In the field of neuroscience, homeostasis has important implications for maintaining normal brain development and function. The brain circuits are formed by different cells connecting to each other, and synaptic communication between them is highly dynamic and activity-dependent. At the individual neuron level, the overall output (i.e., firing rate) of any given neuron is tightly regulated by internal feedback loops to regulate its intrinsic excitability (e.g., voltage-gated ion channels) and by external inputs (e.g., glutamatergic and GABAergic) from other cells in the network to achieve equilibrium (or setpoint). The maintenance of the synaptic balance at a specific setpoint between neurons can be described as synaptic homeostasis, where the excitation of the cells is counteracted by inhibition in brain circuits.

Previous studies have identified that the hippocampus homeostatic setpoint is regulated by DHODH (Styr et al., 2019; Ruggiero et al., 2021), an enzyme residing in the inner membrane of mitochondria and facilitating the electron transfer from dihydroorotate to ubiquinone. Specifically, DHODH inhibition reduced the firing rate in hippocampal neuronal culture measured by MEA plate, which stayed at a reduced rate days after inhibition, resetting a homeostatic setpoint for the neurons. The studies implicated a metabolic substrate underlying the firing setpoint, providing an explanation for the chronic development of epilepsy. Although the relation between mitochondria and the mTOR pathway in the brain remains unclear, studies have revealed that rapamycin treatment inhibiting the mTOR pathway led to changes in mitochondrial protein phosphorylation in human T lymphocyte cells (Betz and Hall, 2013). These studies allude a critical role of mitochondria in homeostasis, which could be modulated by mTOR activity.

### Synaptic Homeostasis in Genetic Epilepsy

In normal development, homeostasis is maintained at a setpoint value, while in the case of epilepsy, homeostatic plasticity is disrupted with an elevated setpoint of global excitation over inhibition due to maladaptive changes, such as mutations of signaling molecules along the mTOR pathway or ion channels underlying intrinsic excitability or synaptic proteins. As discussed above, changes of ion channels in excitatory neurons, such as LOF in KCNQ2 or GOF in KCNT1, can lead to an increase in intrinsic excitability and excessive firing from the neurons. Other ion channel mutations, such as SCN1A in the inhibitory neurons, can lead to decreased excitability, allowing disinhibition of the excitatory neurons. Components along the mTOR pathway have also been shown to be a hotspot for epilepsy, reflected by its mutation displaying not only a higher firing rate, but also changes in morphology. Although often overlooked under the context of electrical activity, morphological alteration such as sprouting is closely linked to hyperconnectivity and, in turn, disrupted electrical activity in the brain circuit can lead to epilepsy. While the changes in intrinsic excitability, synaptic activity, and morphology may be parallel and converging, there is no direct evidence with human *in vitro* studies that explicitly parse out primary, secondary and/or compensatory effects. This presents a major challenge for the field to test. It is conceivable that morphological and electrical analysis can be done in a time series experiment to temporally map the changes in intrinsic excitability, synaptic activity, and morphology. Experiments blocking one process or another at different time points with pharmacological blockers or genetic perturbations will help clarify their intricate relationships and reciprocal dependence.

Both ion channel and the mTOR pathway mutations are tightly related with synaptic dynamics and epileptic activity, and these two groups of mutations might be associated with each other. While one paper has discovered ion channel deficit (KCNQ2) in UBE3A human *in vitro* model, the underlying connection between the ion channel and the mTOR pathway has rarely been explored in the human *in vitro* model, it has been examined in mouse podocytes, where mTOR was found to regulate intrinsic excitability by increasing calcium-activated potassium channels (BK channels) expression and BK channel conductance was decreased when the pathway was inhibited by AKT inhibitor (Wang et al., 2019). Interestingly, the mTOR pathway can also directly regulate ion channels and synaptic proteins. For example, NMDAR recruits PTEN onto postsynaptic density and decreases AMPAR mediated responses (Jurado et al., 2010). In addition, PTEN knockdown in iPSC-derived motor neurons has been shown to alter the properties of AMPARs directly, such as their expression level and activity reflecting synaptic strength and transmission (Yang et al., 2014). Ion channels and the mTOR pathway can both regulate synaptic plasticity, remodeling the dynamics between individual synapses and the setpoint of neurons and networks. In other studies using mice and rat models (Niere and Raab-Graham, 2017), mTOR inactivation elevated Kv1.1 and Kv1.2 expression and regulated NMDAR activity, which controlled Ca^2+^ influx into the cell to alter mTOR activity. This self-regulated feedback loop maintains cellular excitability by balancing the changes in ion channels and mTOR activity, likely through excitation-transcription coupling. More importantly, a study directly examined the link between ion channel and mTOR pathway under the context of epilepsy (Nguyen and Anderson, 2018). In PTEN KO mice, hippocampal Kv1.1 protein expression was increased, and inhibition of mTOR with rapamycin normalized the aberrant expression. In light of these results, where mTOR has a significant effect on the protein synthesis for Kv1.1, it is possible that mTOR can also alter the translation and activity of a variety of ion channels in the neurons, and thus regulating excitability in the neuronal network. Clarifying the type of neurons associated with Kv1.1 reduction and their impact on the E/I dynamics will unravel its relation to epilepsy in the PTEN KO mice.

**FIGURE 1:**
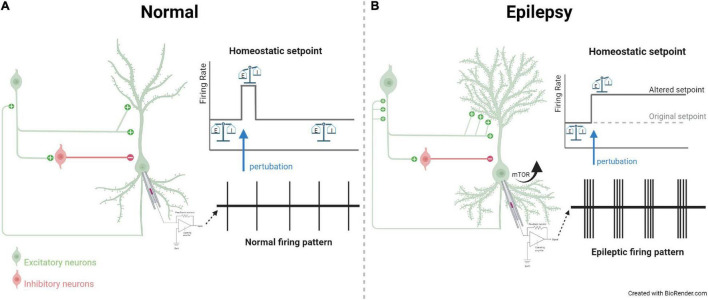
Hypothetical working model contrasting a microcircuit in normal and epileptic brain. **(A)** In normal condition, excitatory and inhibitory inputs synapsing onto a neuron with a balanced E/I ratio and firing pattern. Upon perturbation, homeostatic setpoint resets the electrical activity back to baseline. **(B)** In epilepsy, channelopathy or mTOR signaling mutations can lead to changes in intrinsic excitability and synaptic strength of inputs. These changes lead to E/I imbalance and result in characteristic epileptic electrical activity, as indicated by the firing pattern recorded from extracellular recording. Alterations in mTOR pathway also result in morphological changes, including enlarged soma and increased sprouting, further aggravating an imbalanced neuronal circuit. During such a perturbation, the homeostatic setpoint is altered in epilepsy and the electrical activity is unable to return to the baseline setpoint. The figure is created with Biorender with publication license.

To conceptualize the findings from both human *in vitro* system and rodent models in which the roles of the mTOR pathway in regulating ion channels and synaptic receptors are evidently established, we propose a hypothetical working model that dysregulation of mTOR signaling may underlie the inability for neurons to adjust the setpoint in their intrinsic excitability and synaptic inputs, both of which converge to an E/I imbalance in the network, leading to seizure activity (Figure 1). Furthermore, cells with mTOR mutations have also shown morphological changes, including enlarged soma and increased dendritic spine density, contributing to hyperfiring and hyperwiring in epilepsy. To maintain a functional neural circuit under the dynamic changes to perturbations, the presence of negative feedback to maintain homeostasis is extremely crucial. Under the normal condition, a neuronal network renders a typical firing rate which is maintained at a particular setpoint for homeostasis, possibly facilitated by a metabolic regulator mitochondrial DHODH and its inhibition. When perturbation in the electrical activity (e.g., sudden hyperexcitation or hypersynchrony) is present, the neuronal circuit can sense the change and efficiently make adjustments to return to the original setpoint. In epilepsy, mutations in the mTOR pathway may reset not only cellular excitability and seizures, but also dysregulate mitochondrial function and morphological sprouting, precluding perturbation-induced readjustment of the setpoint from transforming a highly synchronized and excited state back to the norm. Therefore, the mTOR pathway may play an indispensable role in reciprocally coupling of excitation, transcription and translation of key substrates that are important for self-facilitated homeostasis and E/I balance. Mutations along the mTOR pathway converge to changes in intrinsic neuronal excitability and synaptic inputs, aggravating the hypersynchrony in the network and leading to epilepsy.

## Concluding Remarks and Future Direction

Previous animal models have provided important insights into epilepsy, but our lack of understanding in species differences limits our comprehension of epileptogenesis in human brains and the development of effective treatments for patients. In this review, we summarize a series of studies of ion channels in epilepsy using human pluripotent stem cells derived *in vitro* culture, including voltage-gated sodium and potassium channels. Many other ion channels, such as calcium channels and HCN channels, that have been investigated in animal models are yet to be studied in human *in vitro* culture. In addition, synaptic receptors that are important for synaptic plasticity, such as NMDAR and GABAergic receptors, that are implicated in patients and studied in animal models (Oyrer et al., 2018), await systematic studies in human *in vitro* systems. Finally, components along the mTOR pathway, including PTEN, PI3K, AKT that are all reported in epilepsy patients remain underexplored in the context of epilepsy with PSC-derived systems. Many of the studies have used relatively simplified neuronal cultures. As these models serve as a reductionist approach to study epilepsy, it is also essential to acknowledge the crucial roles of interactive dynamics in neurons and neuronal networks. Homeostasis probably requires a highly complex circuit model with excitatory principal neurons and inhibitory interneurons and other cell types including astrocytes, oligodendrocytes and microglia. To achieve this, co-cultures of mixed cell types or brain organoids can be employed to develop a more physiologically relevant network. Other models such as the xenografted mouse model carrying specific human patient cells should be used to provide a native host for these induced neurons to form complex circuits reminiscent of epileptic loci *in vivo*. In doing so, one can address the possibility how mutant neurons prime the seizure and epilepsy with EEG correlate and behavioral outcomes as objective readouts. As proposed in our hypothetical working model, we suggest that the mTOR activity affects ion channels, synaptic plasticity and hyperwiring, which underlies E/I imbalance and permanently altered homeostatic set point, preventing the electrical activity from being renormalized after perturbation. The dynamic interactions between the mTOR pathway, ion channels, and synaptic plasticity have hardly been investigated using human *in vitro* systems, which undoubtedly will offer efficient platforms for us to scrutinize complex brain networks and gain unprecedented insights into homeostatic alterations in epileptogenesis for developing therapies.

## Author Contributions

OW, YL, and L-YW contributed to conception and design of the study. OW wrote the first draft of the manuscript. All authors contributed to manuscript revision, read, and approved the submitted version.

## Conflict of Interest

The authors declare that the research was conducted in the absence of any commercial or financial relationships that could be construed as a potential conflict of interest.

## Publisher’s Note

All claims expressed in this article are solely those of the authors and do not necessarily represent those of their affiliated organizations, or those of the publisher, the editors and the reviewers. Any product that may be evaluated in this article, or claim that may be made by its manufacturer, is not guaranteed or endorsed by the publisher.
